# A Case of Apparent Upper-Body Freezing in Parkinsonism while Using a Wheelchair

**DOI:** 10.3389/fneur.2017.00205

**Published:** 2017-05-15

**Authors:** Samuel T. Nemanich, Marie E. McNeely, Gammon M. Earhart, Scott A. Norris, Kevin J. Black

**Affiliations:** ^1^Program in Physical Therapy, Washington University School of Medicine in St. Louis, St. Louis, MO, USA; ^2^Department of Neurology, Washington University School of Medicine in St. Louis, St. Louis, MO, USA; ^3^Department of Neuroscience, Washington University School of Medicine in St. Louis, St. Louis, MO, USA; ^4^Department of Radiology, Washington University School of Medicine in St. Louis, St. Louis, MO, USA; ^5^Department of Psychiatry, Washington University School of Medicine in St. Louis, St. Louis, MO, USA

**Keywords:** Parkinson’s disease, hypokinesia, upper-limb freezing, freezing, akinesia

## Abstract

Freezing of gait (FOG) is a common, disabling gait disturbance in Parkinson’s disease (PD) and other Parkinsonian syndromes. Freezing also occurs during non-gait movements involving the upper limbs. The mechanisms underlying freezing are complex, likely involving motor, cognitive, and sensory systems that contribute to the episodes. Here, we reported a 60-year-old female with a 24-year history of parkinsonism who experienced significant FOG when ambulatory. Disease progression resulted in her permanent use of a powered wheelchair. While using the power chair, the patient experiences apparent paroxysmal freezing in the hand and arm used to steer and propel the chair. These episodes, some lasting up to several minutes, occur only in circumstances (e.g., entering and leaving an elevator) that are similar to environments known to elicit and exacerbate FOG. Episodes are transient and can be volitionally interrupted by the patient but sometimes require external assistance. Therapeutic intervention for this type of potential freezing has yet to be determined. This case may provide insight into the complex nature of freezing behavior and suggests a need for new approaches to treating non-traditional freezing behavior.

## Introduction

Freezing of gait (FOG) is a common and disabling motor symptom of Parkinson’s disease (PD) and other Parkinsonian conditions. FOG is defined as a “brief, episodic absence or marked reduction of forward progression of the feet despite the intention to walk” (1). Approximately half of people with PD will experience FOG as the disease progresses (2). While it is typically associated with greater disease duration and dose of levodopa medication, FOG can affect some individuals earlier in the disease course (3). The health-related impact of FOG is concerning. FOG is associated with an increased risk of falling (4, 5), reduced quality of life (6), and cognitive impairment (7, 8). While pharmacologic and surgical treatment can alleviate freezing episodes in some patients, others continue to exhibit freezing behavior despite optimal treatment with these therapies (9, 10). These observations suggest that FOG may involve non-dopaminergic systems (3), but the causal pathways remain poorly understood.

While freezing predominantly affects walking and stepping, recent work showed that freezing can affect non-gait motor systems as well (11). These data indicate that similar freezing can occur during upper-limb and finger movements (12–14), as well as during speech (15). While gait and non-gait freezing do not necessarily co-occur, these data suggest that some common mechanism underlies motor arrests in PD. As such, in the remainder of this case report, we will use the term “freezing” to include both gait and non-gait phenomena.

Various hypotheses describing potential mechanisms of freezing have been proposed, including impaired automaticity (16), perceptual impairments (17), and cognitive-motor decoupling (18), and this remains an active area of research. Furthermore, non-dopaminergic systems such as serotonergic neurons in the raphe nucleus and cholinergic neurons in the pedunculopontine nucleus may also play a role (3). Thus, freezing likely involves a widespread network of various brain regions. In this case report, we describe a patient with dopa-responsive parkinsonism who experienced gait freezing when ambulatory and currently experiences apparent freezing only when operating her powered wheelchair in situations that often elicit gait freezing. The purpose of this case is to present a novel task-specific case of potential freezing and to summarize and expand on the potential neurological mechanisms of freezing.

## Case Report

The patient is a 60-year-old woman with a 24-year history of parkinsonism currently residing in an assisted living facility. She initially presented to our movement disorder clinic in 1996 with a 4-year history of gradually progressive lower extremity pain and spasms, and blepharospasm following constipation and urinary urge. Baclofen and botulinum toxin targeting ocular muscles provided modest benefit for spasms. The differential remained broad and initial workup included normal brain magnetic resonance imaging (MRI), cervical, thoracic, and lumbar spine MRI, cerebrospinal fluid studies (cell count, protein, glucose, oligoclonal bands, Lyme titers), complete blood cell count, comprehensive metabolic panel, thyroid-stimulating hormone level, triiodothyronine (T3) levels, free thyroxine (T4) levels, serum immunofixation, ceruloplasmin, B12, Venereal Disease Research Laboratory, erythrocyte sedimentation rate, extractable nuclear antigen, antinuclear antibody (slightly elevated, but rheumatology follow-up revealed no association with clinical syndrome), vitamin E, human immunodeficiency virus, human T-cell lymphotropic virus, very long chain fatty acids, electromyography/nerve conduction studies, serum and urine amino acids, conjunctival biopsy for lipofuscinosis, urine samples for arylsulfatase A, negative gene testing for spinocerebellar ataxia-type 3, and Huntington disease.

Over the next 2 years, she displayed progressive asymmetric (left more than right) leg and arm dystonia with concomitant development of asymmetric leg then arm rest tremor, rigidity, and impaired gait (asymmetric foot dragging) and balance. PD was diagnosed in 1998 following exquisite improvement of rest tremor, rigidity, dystonia, gait, and blepharospasm with initiation of carbidopa/levodopa (Unified Parkinson’s Disease Rating Scale motor subsection (UPDRS III) score improved from 38 to 15.5 with titration). Over the next several years, she developed motor fluctuations with painful OFF-state left arm/leg dystonia and peak-dose cervical dystonia (worse on the left). Trials with ropinerole provided initial relief of OFF-state dystonia, but in late 2001, she developed dose-limiting peak-dose dyskinesia and was unable to tolerate ropinerole or trials with pergolide, pramipexole, tolcapone, or entacapone in addition to amantadine and levodopa. Over the next 3 years, she experienced worsening gait (due in part to worsening left leg dystonia) with freezing that resulted in at least 2 falls per week by 2004. In 2006, on examination, gait impairment was characterized independent of dystonia by inability to lift her feet, particularly on the left side, resembling the akinetic subtype of FOG (19). At this time, FOG was severe enough that she used a wheelchair when outside of the home for safety.

In June 2006, she underwent successful implantation of bilateral subthalamic nucleus deep brain stimulation after OFF/ON evaluation revealed improvement of the UPDRS III score from 69 in the practically defined OFF state to 13.5 following ingestion of 600 mg levodopa. DBS resulted in markedly reduced rigidity and tremor, and relieved pain secondary to left arm and leg dystonia which allowed reduction of levodopa dosing over 50% during the first year. After DBS, the patient’s gait and mobility improved substantially, and she did not regularly use her power scooter. Three years following DBS, she required dose titrations and again developed motor fluctuations with worsening rest tremor, dystonia, and FOG. By July of 2009, she was still able to walk, but again preferred to use a power chair due to fear of falls in the context of worsening FOG. She was bound to her power chair in 2012 due to progression of gait instability and FOG with increased motor fluctuations.

In April 2015, the patient first reported intermittent right hand freezing while controlling her power wheelchair in specific situations. Three months later, this apparent freezing of the upper extremity began to interfere substantially with her ability to propel her power wheelchair, particularly when entering an elevator or passing through doorways. The apparent freezing was mostly in her fingers, which controlled the movement of her chair. During episodes, the patient does not display dystonic posturing of the wrist or hand typical of her OFF-period dystonia, or as an overflow phenomenon. As reported in May of 2016, the navigation-related upper-limb freezing episodes occur a few times per day. The suspected freezes observed in the clinic usually lasted just a few seconds, but the patient reports episodes may last for as long as 1–10 min. The patient is typically able to break the motor arrests using her other hand (left) to release fingers of the right hand from the steering knob. In cases where the episodes last several minutes, staff members at her living facility intervene to help break the motor arrest. These apparent freezing episodes occur when moving the wheelchair both forward and backward. Entering and exiting elevators are the most common triggers, though navigating around the dining room in the presence of other residents who make her feel nervous occasionally triggers these episodes of potential freezing. The patient reports that these navigation-related upper-limb freezing episodes are similar to gait freezing she experienced previously and are accompanied by similar sensations and feelings of panic without other panic attack features. These episodes can occur either at peak dose or in the off state, but are more frequent and last longer in the off state. Because the patient is no longer ambulatory, we cannot determine if she still experiences gait freezing. The patient does not report this potential upper-limb freezing during any other upper-limb tasks nor does the apparent upper-limb freezing occur during wheelchair steering in other circumstances, such as navigating a wide open space. This is consistent with observations in our clinic.

On examination, she had saccadic intrusions on smooth pursuit, but no additional extraocular movement abnormalities. Finger tapping, hand movement, and leg agility were slow with diminished amplitude in the OFF more than ON state, but movement always remained visible, except at the moments described when operating her chair. Bradykinesia remained fairly constant in the OFF state, whereas the apparent freezing phenomenon was transient, most often lasting about 5–15 s (though occasionally longer). There was no increase or change in dystonic posturing when she attempted to initiate hand movements to control the wheelchair, and she lacked subjective pain that frequently accompanied OFF-period dystonia. She was otherwise able to accurately maneuver the wheelchair throughout most of the day, despite obvious and painful hand dystonia. She denied subjective concerns of not knowing how to make the desired movement during freezing episodes, and she lacked constructional or ideomotor apraxia on formal testing. Freezing episodes did not depend on whether observers were present or on any apparent psychological rewards.

She has an extensive psychiatric history including the following symptoms: major depression, hallucinations, and suicidal ideation. Psychiatric treatments have included thiothixene, quetiapine, and electroconvulsive therapy with good benefit. Antipsychotics were last prescribed 10–15 years prior to her first visit at our center (except for 1–3 nightly doses of quetiapine 12.5 mg in 2014). Depression occurring concomitantly with parkinsonism was treated intermittently with nortriptyline, mirtazapine, paroxetine, nefazodone, and duloxetine. The patient has a family history of paranoid schizophrenia (brother) and alcoholism. At present, she is cognitively normal (Mini-Mental Status Exam score of 27, Montreal Cognitive Assessment score of 28) and has no evidence of changes in memory.

The patient’s current medications as of October 2016 are summarized in Table 1, categorized as “parkinsonism,” “psychiatric,” and “pain.” Her prescribed levodopa equivalent daily dose is 650 mg, and she currently takes only levodopa (i.e., no agonists or MAOB inhibitors). Frequent revision of medication schedules, particularly following DBS, was a major part of her management as the patient experienced constant debilitating pain, frequent on- and off-period dystonia, and occasional hallucinations. Motor benefit from levodopa (taken every 3 h from 6 a.m. to 6 p.m. and CR at 9 p.m.) appeared in 30 min, and lasted until 30–45 min prior to her next dose. The UPDRS was administered 53 min after a regular dose of carbidopa/levodopa during her October 2016 visit. The summary scores are as follows: Part I: 3/16, Part II: 25/52, Part III: 64.5/108, and Part IV: 4/23. For subscale III (motor), the patient has severe bradykinesia and hypokinesia, hypophonia, and cannot rise from her motorized chair without assistance. DBS settings were last adjusted in January of 2015. Neither medication nor DBS therapies currently provide sufficient relief for this patient’s apparent task-specific upper-limb freezing during navigation, though she reports worse and more frequent episodes during off-medication periods.

**Table 1:** **Current medications**.

	Medication	Total daily dose (mg)	Comments
Parkinsonism	Sinemet (carbidopa–levodopa)	150/600	
	Sinemet (carbidopa–levodopa)	25/100	1 tablet 4×/day, as needed
	Sinemet (carbidopa–levodopa)	50/200	1 tablet at bedtime
Psychiatric	Cymbalta (duloxetine)	90	
	Dextroamphetamine Sulfate	40	
	Mirtazapine	30	
Pain	Acetaminophen	325	As needed
	Baclofen	50	10 mg 3×/day, 20 mg at bedtime
	Gabapentin	3,600	Additional 300 mg as needed
	Ibuprofen	2,400	

## Discussion

Freezing is a complex and poorly understood feature of PD. The pathophysiological and behavioral mechanisms associated with freezing likely involve motor, cognitive, and sensory factors (20). How these factors interact is still not fully understood and, as a result, the current array of treatment approaches for freezing do not provide sufficient long-term benefit (21). In this report, we describe a novel case of a patient who previously experienced gait freezing, and while now non-ambulatory, experiences episodes in the upper limb that appear to be freezes when navigating her wheelchair in specific situations that also commonly elicit FOG. The circumstances and sensations during these episodes as reported by the patient are similar to those experienced during previous gait freezing. To our knowledge, this is one of the first reports of apparent freezing of another part of the body during a navigation-related task (i.e., steering a wheelchair). Systematic studies of upper-limb freezing manipulated the spatiotemporal components of the task to induce freezing (12–14); however, it was not clear if these participants also experienced upper-limb freezing during other tasks or in specific environments (such as when passing through narrow spaces).

While the motor demands differ between walking and navigating a wheelchair, sensory aspects such as optic flow are similar. One current hypothesis is that freezing is due to perceptual impairments related to visuomotor integration of one’s body within the environment (17). Evidence for this comes from patient reports as well as experiments examining how people with PD walk through spaces with varying widths, such as doorways (22, 23). The patient reported that her freezing was worse when entering/exiting elevators and when finding a seat in the dining hall. Both situations challenge spatial judgment, and navigating in the dining hall was also emotionally taxing for this particular patient. In addition, the cognitive and emotional demands associated with these scenarios are similar in both walking and using a wheelchair. Complex tasks can exacerbate freezing (24), and fear related to prior freezing events may increase anxiety and also contribute to freezing (25). This patient has a history of mood disorder that may contribute to emotion-related freezing triggers. Since cognitive-motor overload is also a plausible mechanism for freezing behavior (18), cognitive interference may play a role in this case as it does in other types of freezing. Though we saw no evidence of cognitive impairment in this individual, increasing cognitive load has been shown to trigger freezing episodes in people with PD who experience freezing, regardless of cognitive status (3, 26). As a result, cognitive-motor interference may contribute to this phenomenon. Overall, these hypotheses suggest a more general mechanism that could apply to both FOG and upper-limb freezing during navigation, even though these two tasks have very different motor demands.

We cannot rule out other explanations for the phenomenon described in this case. Dystonia is a common side effect of dopaminergic treatment, usually occurring in the off-period (27). The patient experienced previous dystonia in the lower limbs during both on- and off-medication periods. The interruption in her intended upper-limb movements could also potentially be attributed to a form of dystonia. However, this is unlikely because the patient’s previous dystonia was generalized and not associated with certain tasks or situations, whereas the current apparent freezing occurs only in the upper limb involved in wheelchair navigation and only during specific situations. More directly, dystonic posturing and pain were absent during the episodes, and finger torsion or deviation was not observed upon examination. Further, the patient did not report focal muscle tension during these episodes of suspected freezing while using the wheelchair. Recordings of muscle activity during upper-limb freezing episodes were not able to be collected for this patient. However, in future cases of suspected task-specific upper-limb freezing, this may help distinguish between dystonia, which is characterized by sustained agonist/antagonist co-contraction (28), and freezing, which is typically accompanied by inappropriate timing of agonist/antagonist muscle pairs (3). General but severe bradykinesia could feasibly contribute to the interruption of her upper-limb movements as well. On the UPDRS III, the patient scored a 4 on body bradykinesia and hypokineisa, and a 3 or 4 on finger tapping, hand movements, and leg agility. However, the duration of the episodes are too long to be purely bradykinetic in nature, and the patient is able to volitionally break the motor arrests herself in most cases, similar to what is seen in FOG.

This phenomenon of apparent upper-limb freezing is also unique from typical episodes of lower limb or gait freezing. For example, the maximal duration of the episodes reported by the patient is atypically long compared to most reports of FOG. Most FOG episodes last less than 10 s and rarely last more than 30 s before movement is regained (19). However, episodes observed within the clinic were all within the typical reported range. The lack of arm trembling concurrent with the episode is also uncharacteristic of FOG. However, there are numerous reports of upper-limb freezing episodes described as a cessation of movement in one or more hand that are not accompanied by trembling (29–32). Further, striking similarities between this phenomenon of apparent upper-limb freezing and FOG include the identifiable environmental triggers (crowded spaces, passageways) and the ability to interrupt the episodes.

## Conclusion

Overall, unique cases such as this highlight the need to consider freezing as a problem affecting the entire sensorimotor system and emphasize the need for clinicians and researchers to develop new treatments for non-gait freezing.

## Ethics Statement

The patient gave signed consent for releasing her health information in the format of this case report. The authors consulted the Washington University Human Research Protection Office (IRB), which judged that additional ethics review was not required.

## Author Contributions

STN and MM wrote the manuscript. SAN and KB collected data. STN, MM, GE, SAN, and KB edited the manuscript.

## Conflict of Interest Statement

The authors declare that the research was conducted in the absence of any commercial or financial relationships that could be construed as a potential conflict of interest.
